# ABCB1 confers resistance to carboplatin by accumulating stem-like cells in the G2/M phase of the cell cycle in p53^null^ ovarian cancer

**DOI:** 10.1038/s41420-025-02435-7

**Published:** 2025-04-02

**Authors:** Danbi Lee, Hyun-Seok Jeong, Sun-Young Hwang, Yu-Gyeong Lee, Youn-Jung Kang

**Affiliations:** 1 Department of Biomedical Science, School of Life Science, CHA University, Seongnam-si, South Korea; 2 Department of Biochemistry, Research Institute for Basic Medical Science, School of Medicine, CHA University, Seongnam-si, South Korea

**Keywords:** Ovarian cancer, Translational research, DNA damage checkpoints

## Abstract

High-grade ovarian serous carcinoma, mostly bearing the various mutations in the *TP53* gene, typically relapses within six months after first-line therapy due to chemoresistance, with a median overall survival of less than a year. However, the molecular mechanisms of action behind acquired drug resistance, particularly in relation to different *TP53* mutation types, have not been fully elucidated. In this study, we demonstrated that acquired resistance to carboplatin in SKOV3 harboring a p53^null^ mutation, but not in OVCAR3 with a p53^R248Q^, induces a significant portion of cells accumulated in the G2/M phase of the cell cycle, where cells highly expressed stemness marker with elevated proliferative capacity, which we believe was reversed by ABCB1 inhibition to the levels observed in non-resistant parental cells. ABCB1 suppression re-sensitized carboplatin-resistant cells to additional genotoxic stress and reduced their proliferative ability by recovering DNA repair activity and lowering stemness-like features, especially in the G2/M-distributed fraction. This suggests that high levels of stemness and attenuated DNA repair function exhibited in the G2/M-accumulated portion may be a key contributor of chemoresistance in patients with ovarian cancer bearing a p53^null^ mutation, but not other types of mutations expressing p53. Furthermore, the inhibition of ΔNp73 resulted in the suppression of ABCB1, which consequently restricted cell growth in carboplatin-resistant SKOV3, suggesting that the ΔNp73 may act as an upstream regulator of the ABCB1. Notably, combinatorial treatment of carboplatin with the p53 reactivator, APR-246, proved effective in overcoming chemoresistance in OVCAR3 with the p53^R248Q^. Our findings suggest that the ΔNp73-ABCB1 axis is a promising molecular target for carboplatin-resistant ovarian cancers harboring p53^null^ mutations, which we uncovered could be utilized to increase the efficacy of conventional anti-cancer therapies, to develop more efficient combinatorial therapeutic interventions directed toward overcoming the chemoresistance and improving the survival rates in patients with ovarian cancer.

## Introduction

Ovarian cancer is one of the most dreadful gynecologic malignancies worldwide with the worst prognosis and the highest mortality rates [1]. Despite advances in diagnostic techniques and new therapeutic regimens for this disease, the 5-year survival rate of patients with advanced-stage ovarian cancer still remains around 30%, which is primarily due to late detection of the disease and chemoresistant profiles occurring after an initial good response to anti-cancer treatments [2, 3]. Platinum-taxane-based combinatorial chemotherapy primarily using carboplatin or paclitaxel, which aims to regulate the aberrant cell cycle leading to tumor cell death, is the gold standard of care for patients with ovarian cancer who undergo debulking surgery [4]. However, many cases of high-grade ovarian serous carcinoma, the most common subtype of ovarian cancer, mostly bearing the various mutations in the *TP53* gene, relapse within 6 months after the first-line therapy due to chemoresistance, with a median overall survival of less than a year [5, 6].

The *TP53* gene is a well-known nuclear transcription factor that functions as a tumor suppressor and a master regulator of a variety of target genes, regulating their biological processes, including apoptosis, cell cycle arrest, senescence, and DNA repair [7]. One of the major roles of p53 is to regulate multiple cell cycle checkpoints during the G1/S or G2/M transition phase, which determines the cell fates for DNA repair or programmed cell death [8]. Platinum-based chemotherapy induces intra- or inter-strand crosslinks that activate DNA damage response signals, which enforce cell cycle arrest and subsequently undergo either DNA repair or apoptosis in cells presenting with unrepairable drug-induced DNA lesions [9]. Upon DNA damage, wild-type p53 is activated, stimulating the transcriptional regulation of the downstream cell cycle regulatory protein, p21, which contributes to cell cycle arrest by acting as a cyclin-dependent kinase (CDK) inhibitor. The activity of the CDK1/cyclin B complex is inhibited, resulting in cell cycle arrest at the G2/M phase, where cells frequently stop proliferating and undergo apoptosis [10, 11]. However, p53 mutation-mediated loss of checkpoint regulation and defects in surveillance pathways loosen the control of cell proliferation, leading to the propagation of damaged DNA into their progenies and transformation into cancerous cells [12]. Mutations in the *TP53* gene reported to occur in 62% of high-grade ovarian serous carcinomas are distributed in all coding exons with a strong predominance in the DNA-binding domain region (referred to as hotspot residues in between of 94 and 292), which results in the abrogation of the sequence-specific DNA binding by p53 [13, 14]. These mutations have become more focused in cancer-related studies for a number of clinically relevant reasons. The correlation between p53 mutation status and sensitivity to anti-cancer drugs has been largely investigated, revealing a more frequent occurrence of resistance to cisplatin or carboplatin chemotherapy in patients with p53 mutations than in those with wild-type p53 [14]. However, the molecular mechanisms of action, through which drug resistance is frequently acquired within the correlation between individual *TP53* mutation types and their mediation of aberrant cell cycle progression, are poorly understood [15].

In this study, we demonstrated that carboplatin-resistant p53-null ovarian cancer cells (SKOV3; p53^H179R^) are highly accumulated in the G2/M phase of the cell cycle, displaying an elevated level of ABCB1 together with elevated stemness-related gene expression and highly proliferative properties compared to non-resistant SKOV3 and carboplatin-resistant p53-expressing cancer cells (OVCAR3; p53^R248Q^). Suppression of stem-like property of the G2/M-distributed carboplatin-resistant SKOV3 via inhibition of ABCB1 re-sensitizes carboplatin-resistant SKOV3 to genotoxic stress, displaying a recovery of the DNA damage response and repair function to the levels observed in non-resistant parental cells. Thus, aberrant accumulation in the G2/M phase of the cell cycle with high levels of stemness-related gene expression may be a critical cause of chemoresistance in patients with ovarian cancer bearing a p53^null^ mutation. Understanding the interplay between the G2/M accumulation and induced stemness within the strong correlation of upregulated ABCB1 in carboplatin-resistant p53^null^ ovarian cancer cells is crucial for developing new therapeutic strategies aimed at overcoming resistance and improving patient outcomes.

## Results

### Acquired resistance to carboplatin renders more stemness-like features in p53^null^ human ovarian cancer cells

**Fig. 1:**
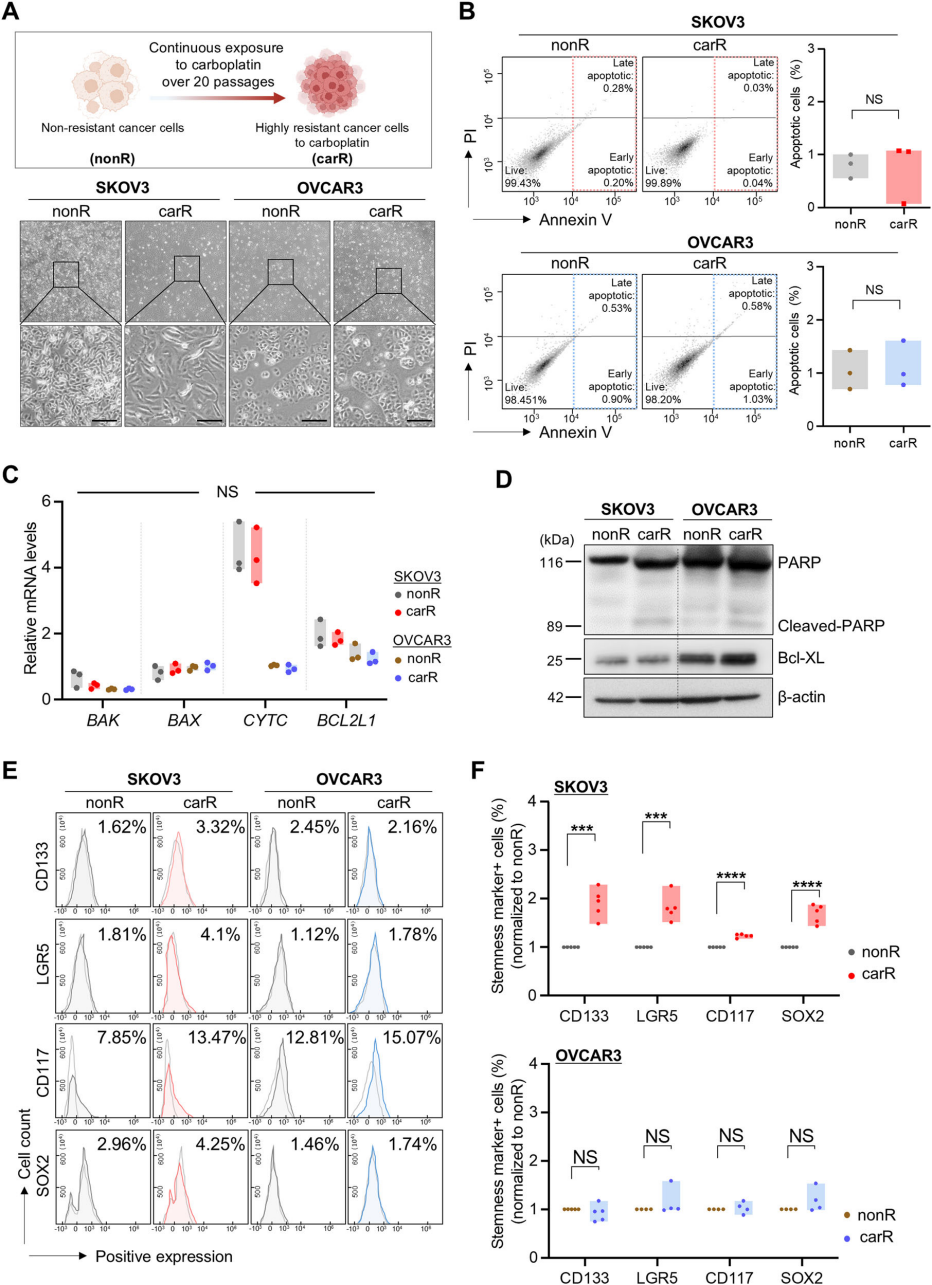
Characterization of carboplatin-resistant p53^null^ SKOV3 compared to p53^R248Q^ OVCAR3. **A** A schematic diagram of establishing the carboplatin-resistant human ovarian cancer cell lines and representative morphological images of each cell line. Scale bar: 25 μm. **B** Cell apoptosis assay of carR-SKOV3 and OVCAR3 compared to each nonR group (SKOV3, *p* = 0.8694; OVCAR3, *p* = 0.8192). Quantified apoptotic level of each group was displayed in graphs on the right. **C** QRT-PCR analyses of apoptosis-related genes (*BAK*, *BAX, CYTC*, and *BCL2L1*) in carR-SKOV3 and -OVCAR3 compared to nonR groups, respectively. **D** Immunoblotting analyses of apoptosis-related proteins (PARP, Cleaved-PARP, and Bcl-XL) in carR-SKOV3 and -OVCAR3 compared to each nonR group. Loading control: β-actin. **E** Stemness profiling of carR-SKOV3 and -OVCAR3 compared to nonR groups, respectively (SKOV3 nonR vs carR: CD133, *p* = 0.0002; LGR5, *p* = 0.0002; CD117, *p* = 0.0001; SOX2, *p* = 0.001/OVCAR3 nonR vs carR: CD133, *p* = 0.9255; LGR5, *p* = 0.5808; CD117, *p* = 0.9733; SOX2, *p* = 0.3877). Quantified positive cell proportions of stemness-related markers (CD133, LGR5, CD117, and SOX2) were displayed in graphs of (**F**). (NS, *p* > 0.05; *p < 0.05; **p < 0.01; ***p < 0.001; ****p < 0.0001).

To generate carboplatin-resistant ovarian cancer cell lines, cells were continuously exposed to carboplatin (SKOV3; 2.5 μM, OVCAR3; 0.25 μM) over 20 passages (approximately 6 months) as previously described [16]. The concentration for each cell line was determined by IC_50_ values and the tolerance rates [17]. SKOV3, harboring a point mutation of H179R in the *TP53* gene, showed no p53 expression at the protein level; thus, they were considered p53^null^, whereas OVCAR3 containing an R248Q point mutation in the *TP53* gene expressed both total and phospho-p53 proteins, displaying an increasing pattern of phospho-p53 expression when exposed to additional carboplatin-induced extra DNA damage (Supplementary Fig. 1A). Carboplatin-resistant SKOV3 (carR-SKOV3) cells exhibited morphological changes displaying more elongated and spindle-shaped cells with decreased *CDH1* and increased *CDH2* expression, which are key features of mesenchymal cells, compared to non-resistant cells (nonR); this change in morphology was not observed in carboplatin-resistant OVCAR3 (carR-OVCAR3) (Fig. 1A and Supplementary Fig. 1B). Moreover, as previously demonstrated, acquired resistance to carboplatin renders more proliferative and migratory ability in SKOV3 compared to OVCAR3 (Supplementary Fig. 1C–E). This was supported by the significantly higher viability in response to an additional carboplatin treatment at 72 h in carR-SKOV3 and carR-OVCAR, compared to each nonR group, implying that carboplatin-resistant cells were more tolerable to additional genotoxic stress (Supplementary Fig. 1F). Of note, carR-SKOV3 with truncated p53 function showed a higher level of tolerance to additional drug treatment than p53-expressing carR-OVCAR3. The concentration of additional carboplatin required to induce extra DNA damage was 10-fold higher than the concentration used to establish a carboplatin-resistant cell line. Indeed, there was no significant difference in the Annexin V-positive apoptotic portion of carR compared to nonR, consistent with the mRNA levels of apoptosis-related genes (*BAK*, *BAX*, *CYTC*, and *BCL2L1*) and the protein levels of common apoptosis-related markers (total PARP, cleaved-PARP, and BCL-XL) (Fig. 1B–D).

It is well recognized that stemness is a major factor that determines the capability of tumor initiation, metastasis, and drug resistance in various cancers, including ovarian cancer [18]. To examine the correlation between the stemness features and acquired drug resistance depending on the status of the p53 mutation in ovarian cancer cells, we estimated the stemness-like properties of carR-SKOV3 and -OVCAR3 cells compared to each nonR control group (Fig. 1E, F). Flow cytometry analyses revealed that acquired resistance to carboplatin significantly increased the levels of stemness-related proteins in SKOV3, whereas no significant difference was observed between carR-OVCAR3 and nonR-OVCAR3. This implies that the level of attained stemness-like properties accompanied by acquired drug resistance is associated with the status of mutations in the *TP53* gene in ovarian cancer cells.

### Carboplatin resistance accumulates p53^null^ ovarian cancer cells in the G2/M phase of the cell cycle, displaying higher stemness features

**Fig. 2:**
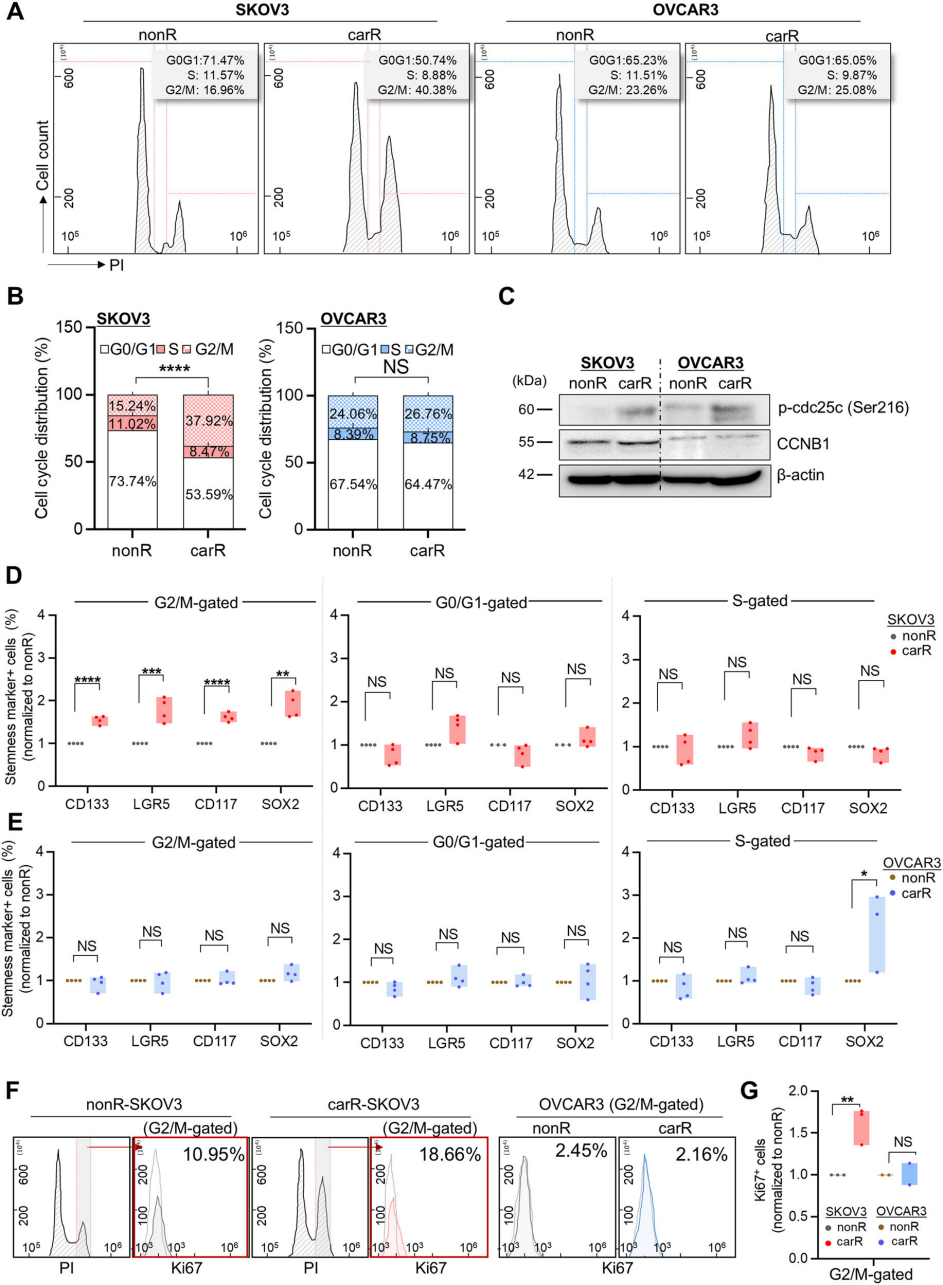
Accumulation of carboplatin-resistant SKOV3 in the G2/M phase of the cell cycle displaying high stemness-like features. **A** Cell cycle analysis in carR-SKOV3 and -OVCAR3 compared to each nonR group. (SKOV3: nonR; *n* = 16, carR; *n* = 13, OVCAR3: nonR; *n* = 10, carR; *n* = 12) Cell cycle distribution in each phase of the cell cycle was shown in graphs (**B**). Statistical analyses were conducted between nonR and carR cells that are distributed in the G2/M phase (SKOV3 nonR vs carR: *p* < 0.001/OVCAR3 nonR vs carR: *p* = 0.0042). **C** Immunoblotting analyses of G2/M transition related-proteins (phospho-cdc25c and CCNB1) in carR-SKOV3 and -OVCAR3 compared to each nonR group. Loading control: β-actin. Analysis of stemness profiling of each phase of the cell cycle in carR-SKOV3 (CD133: 1.55-fold, *p* < 0.001; LGR5: 1.79-fold, *p* = 0.005; CD117: 1.61-fold, *p* < 0.001; SOX2: 1.89-fold, *p* = 0.005) (**D**) and -OVCAR3 (CD133: *p* = 0.512; LGR5: *p* = 0.952; CD117: *p* = 0.839; SOX2: *p* = 0.082) (**E**) compared to nonR groups, respectively. **F** Flow cytometry plots showing the Ki67-positive cell proportion in carR-SKOV3 (*p* = 0.0087) and -OVCAR3 (*p* = 0.9469) compared to each nonR group. Comparison of Ki67-positive proportion in G2/M-gated fraction was displayed in graph (**G**). Data represent the means ± SD from multiple experiments. *p < 0.05, **p < 0.01, ***p < 0.001, ****p < 0.0001, NS not significant.

The differential drug sensitivity of cancer cells is determined by their cellular residence in a specific cell cycle phase, where p53 plays a critical role in the regulation of cell cycle progression [19]. We next examined whether the p53 mutation status affected the distribution of cells across the cell cycle in the presence or absence of drug resistance. Flow cytometry analysis revealed that p53^null^ SKOV3 cells showed significantly higher accumulated proportion in the G2/M phase of the cell cycle as they became resistant to carboplatin. An elevated fraction of G2/M-accumulated cells (23.42%) was accompanied by a decrease in the G0/G1 phase of the cell cycle DNA content of nonR-SKOV3 (20.73%) compared to that in carR-SKOV3 cells. The distribution of carR-OVCAR3 was similar to nonR-OVCAR3 across the entire cell cycle (Fig. 2A, B). This was supported by the increased expression of cell cycle regulator proteins, including phospho-cdc25c (ser216) and cyclin B1, observed in carR-SKOV3 compared to nonR-SKOV3, which was detected with no significant difference between carR- and nonR-OVCAR3 (Fig. 2C and Supplementary Fig. 2A, B). This implicates that the regulation of the cell cycle in response to acquired drug resistance differs from the status of *TP53* gene mutations in human ovarian cancer cells. We then examined the stemness-like profiles of sub-residential cells in each phase of the cell cycle. CarR-SKOV3 cells accumulated in the G2/M phase showed higher increases in the expression of all stemness-related markers compared to nonR-SKOV3. Interestingly, none of the fractions in the G0/G1 and S phases displayed significant changes in SKOV3 cells, and also this was rarely altered in carR-OVCAR3 cells accumulated in the G2/M phase (Fig. 2D). CarR-OVCAR3 cells showed discrepancies in stemness-like properties at each phase of the cell cycle (Fig. 2E). Moreover, Ki67-positive proportion was significantly increased in the G2/M phase of carR-SKOV3, but not in carR-OVCAR3, compared to each nonR group (Fig. 2F, G). Taken together, our findings revealed a subcellular event in which most of the p53^null^ ovarian cancer cells stay longer in the G2/M phase of the cell cycle and attain more stemness-like features when they acquire resistance to carboplatin than p53-expressing ovarian cancer cells.

### ABCB1 is highly expressed in platinum drug-resistant SKOV3 cells

**Fig. 3:**
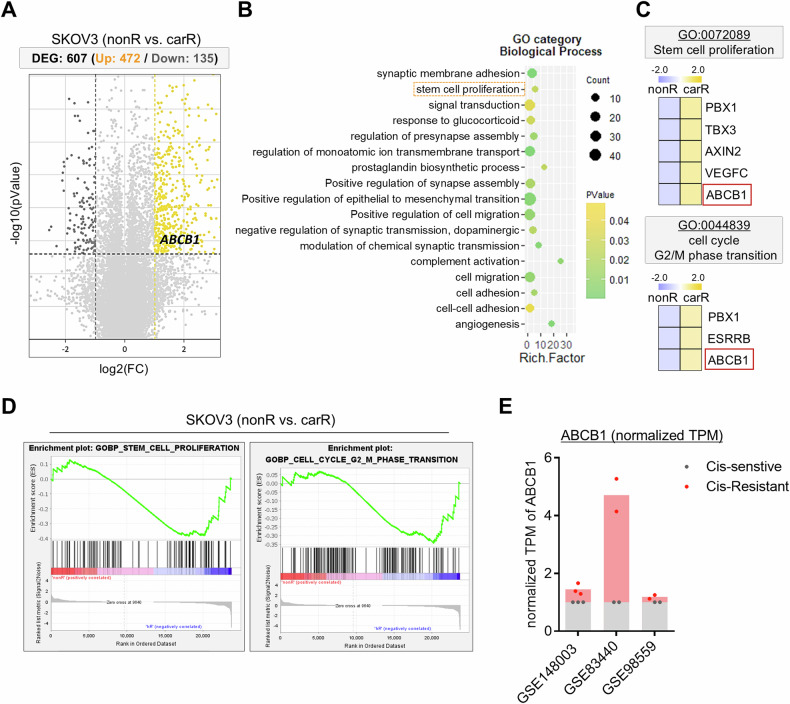
Upregulation of ABCB1 in platinum drug-resistant SKOV3. **A** Volcano plot of DEGs including 472 upregulated genes (yellow) and 135 downregulated genes (dark gray), displaying the ABCB1 gene in the upregulated group. **B** Enriched top 17 pathways obtained from gene ontology (biological process) analysis of DEGs. **C** Heatmap color-coded by *Z*-score showing different gene expression patterns in carR-SKOV3 compared to the nonR group in specific GO terms (GO:0072089 and GO:0044839). **D** GSEA analysis showing enriched biological pathways in carR-SKOV3 compared to nonR-SKOV3. **E** Analysis of datasets (GSE83440, GSE98559, and GSE148003) including upregulated *ABCB1* expression in cisplatin-resistant SKOV3 compared to cisplatin-sensitive SKOV3. Data were quantified by normalized TPM levels of *ABCB1* in each group.

To further evaluate the molecular mechanisms that are involved in the acquired resistance to carboplatin in p53^null^ ovarian cancer cells, we analyzed our previously reported dataset (GSE173579). A total of 607 genes (upregulated: 472 genes, downregulated: 135 genes) were significantly differentially expressed in the carR-SKOV3 group compared to the nonR-SKOV3 group [16]. Among DEGs, the *ABCB1* gene was revealed as one of the genes with increased expression in carR-SKOV3 group (Fig. 3A). Consistently, the level of the *ABCB1* gene was increased in SKOV3 cells when they became resistant to carboplatin, while it remained at a relatively low level in OVCAR3, regardless of the resistance to carboplatin (Supplementary Fig. 3A). Moreover, by analyzing GO enrichment of DEGs, we observed some of denoted signaling pathways, including ‘GO:0072089; stem cell proliferation’ and ‘GO:0044839; cell cycle G2/M phase transition’, in which the *ABCB1* gene was significantly involved, compared to nonR-SKOV3 group (Fig. 3B, C). Gene set enrichment analysis (GSEA) repeatedly showed that carboplatin resistance in SKOV3 resulted in DEG enrichment in the ‘stem cell proliferation’, ‘cell cycle G2/M phase transition’, and ‘epithelial to mesenchymal transition’ pathways (Figs. 3D and S3B), which was consistent with our findings (Figs. 1 and 2). To validate the elevation of the *ABCB1* gene expression in platinum drug-resistant p53^null^ SKOV3 cells, we analyzed public datasets (GSE83440, GSE98559, and GSE148003) and compared cisplatin-sensitive and -resistant SKOV3 cells. As cisplatin is another type of platinum drugs like carboplatin, *ABCB1* expression in cisplatin-resistant SKOV3 cells was expectedly higher than that in cisplatin-sensitive cells (Fig. 3E). Furthermore, the expression of both *CCNB1* and *CDK1* gene was higher in cisplatin-resistant SKOV3 than that in cisplatin-sensitive SKOV3 (Supplementary Fig. 3C). These analyses support our findings that more carR-SKOV3 cells were accumulated in the G2/M phase of the cell cycle than nonR cells (Fig. 2A–C).

### ABCB1-induced stem-like features in G2/M-accumulated cells confer resistance to carboplatin in p53^null^ ovarian cancer

**Fig. 4:**
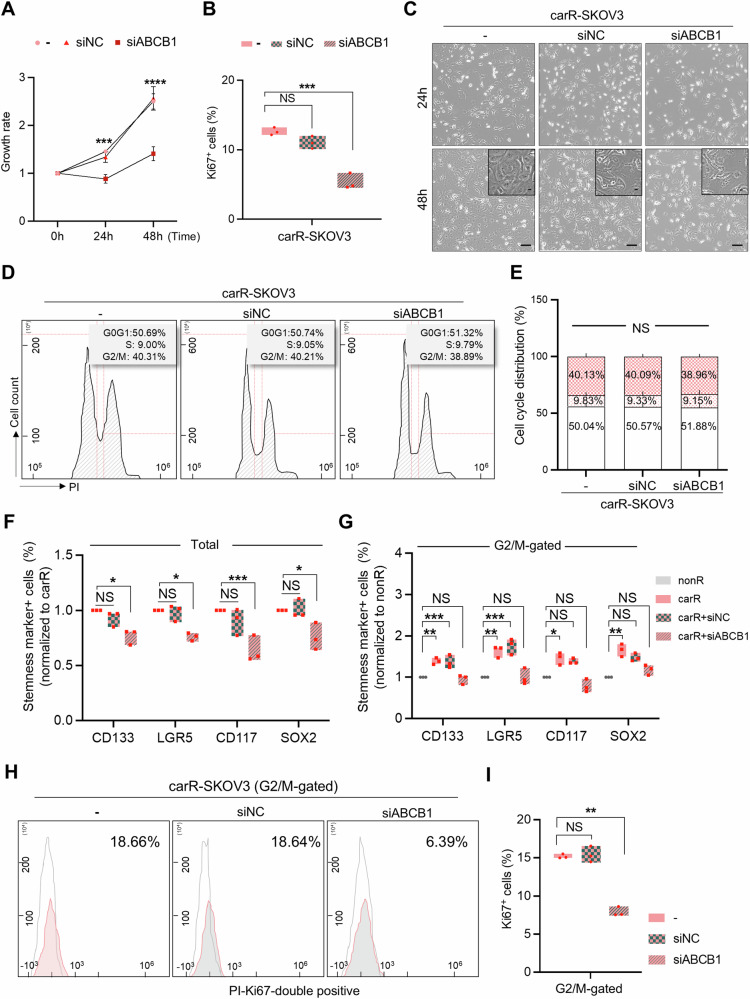
Reduced proliferative rates of carboplatin-resistant SKOV3 by suppression of stem-like features in G2/M-accumulated cells via ABCB1 inhibition. **A** Cell proliferation assays in siABCB1-transfected carR-SKOV3 compared to non-transfected- and siNC(non-targeting)-transfected carR-SKOV3. The percentage of Ki67-positive cell proportion in each group was displayed in a graph (**B**). **C** Morphology of non-treated, siNC-transfected, and siABCB1-transfected carR-SKOV3. Scale bar: 100 μm, 25 μm (magnified images). **D** Cell cycle analysis of non-treated (*n* = 17), siNC-transfected (*n* = 15) and siABCB1-transfected (*n* = 15) carR-SKOV3 group (i) carR vs carR-siNC (non-targeting): G0/G1, *p* = 0.697; S, *p* = 0.601; G2/M, *p* = 0.964; (ii) carR vs carR-siABCB1: G0/G1, *p* = 0.329; S, *p* = 0.126 G2/M, *p* = 0.187; (iii) carR-siNC vs carR-siABCB1: G0/G1, *p* = 0.621; S, *p* = 0.118; G2/M, *p* = 0.135). Cell distribution in each phase of the cell cycle was shown in a graph (**E**). Statistical analyses were conducted between average values obtained from nonR- and carR-SKOV3 cells that are distributed in the G2/M phase. Analyses of stemness profiling of siABCB1-transfected carR-SKOV3 in singlets (CD133, 0.76-fold; LGR5, 0.76-fold; CD117, 0.63-fold; SOX2, 0.75-fold) (**F**) and G2/M phase (CD133, 0.70-fold; LGR5, 0.20-fold; CD117, 0.46-fold; SOX2, 0.76-fold) (**G**) compared to non-treated and siNC-transfected carR-SKOV3. **H**, **I** Flow cytometry plots showing the Ki67-positive cell proportion in G2/M-accumulated siABCB1-transfected carR-SKOV3 compared to non-treated and siNC-transfected carR-SKOV3 groups (*p* = 0.0007). Data represent the means ± SD from triplicate experiments. *p < 0.05, **p < 0.01, ***p < 0.001, ****p < 0.0001, NS not significant.

We previously demonstrated that ABCB1 inhibition re-sensitizes p53^null^ SKOV3 to anti-cancer drugs by recruiting a critical factor of the non-homologous end-joining DNA repair pathway, XRCC4, resulting in the activation of DNA repair activity and reduction of cellular proliferation [16]. In addition to this, in this study we further investigated whether aberrantly upregulated ABCB1 in carR-SKOV3 was responsible for the cellular fraction that is accumulated in the G2/M phase of the cell cycle, displaying a high level of stemness properties within the comparison with OVCAR3, which expressed both p53 and phospho-p53 proteins (Supplementary Fig. 1A). After *ABCB1* knockdown using siRNA transfection, cellular growth rates were significantly reduced, displaying a decreased Ki67-positive portion of cells compared to the control groups, however no morphological changes observed (Fig. 4A–C). Cell cycle evaluation of carR-SKOV3 after *ABCB1* knockdown showed no significant changes in cell cycle distribution compared to the control groups. However, interestingly, the high levels of stemness protein expression in G2/M-accumulated carR-SKOV3 cells were reversed to the lower levels observed in nonR-SKOV3 through ABCB1 inhibition, which might be equivalent to the decrease in stemness protein levels in total carR-SKOV3 cells with ABCB1 inhibition (Fig. 4F, G). Moreover, the Ki67-positive cell proportion in the aberrantly accumulated carR-SKOV3 in the G2/M phase decreased to around a 30% level of the original carR-SKOV3 cells upon ABCB1 inhibition (Fig. 4H, I). Our findings suggest that the elevated stemness-like characteristics in G2/M-accumulated carR-SKOV3 were significantly reduced by ABCB1 inhibition without considering cell cycle progression.

### Oncogenic ΔNp73 drives the resistant phenotype conferred by upregulated ABCB1

**Fig. 5:**
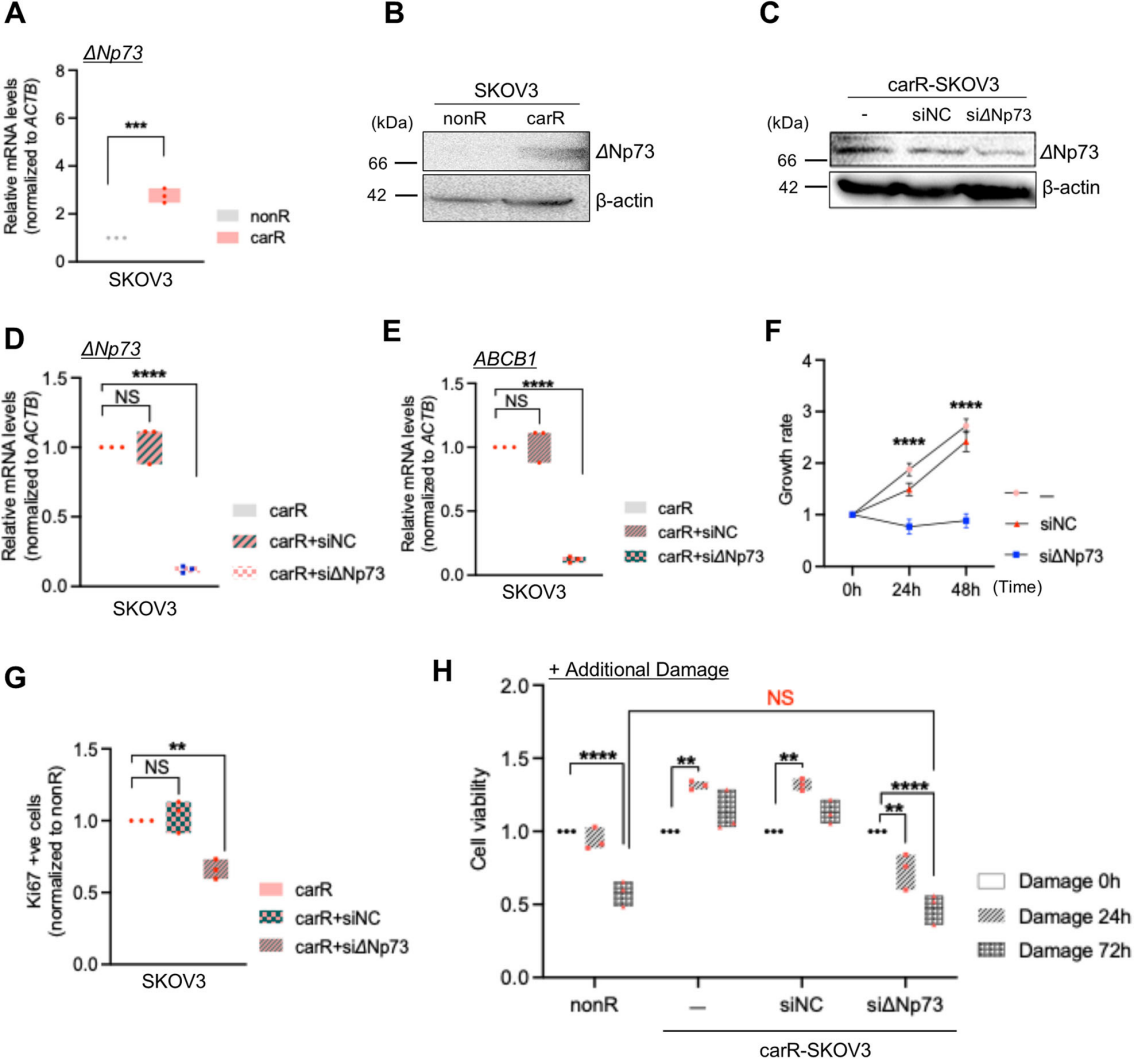
Reversal of the resistant phenotype conferred by upregulated ABCB1 through ΔNp73 suppression. **A** Quantitative gene expression of Δ*Np73* in nonR and carR-SKOV3 cells (*p* = 0.0005). **B** Immunoblotting analysis of ΔNp73 protein levels in nonR and carR-SKOV3 cells via immunoblotting assay. Validation of *Δ*Np73 silencing in carR-SKOV3 compared to non-treated and negative control (NC) transfected-groups both at protein (**C**) and mRNA (**D**) levels (non- vs NC-transfected; *p* = 0.351, non- vs si*Δ*Np73-transfected; *p* = 0.00002). **E** Quantitative gene expression of *ABCB1* in carR-SKOV3 (non-treated, siNC-transfected, and si*Δ*Np73-transfected) cells (non-treated vs siNC-transfected*; p* = 0.468, non-treated vs si*Δ*Np73-transfected*; p* = 0.00005). **F** Relative cell growth rates of siΔNp73-transfected carR-SKOV3 cells compared to non-transfected and NC-transfected cells (24 h: non-treated vs siNC-transfected*; p* = 0.017, 48 h: non-treated vs siNC-transfected*; p* = 0.0002). **G** Comparison of Ki67 expression in carR-SKOV3 (non-treated, siNC-transfected, and siΔNp73-transfected) cells (non-treated vs siNC-transfected*; p* = 0.013). **H** Cell viability assay in carR-SKOV3 (non-treated, siNC-transfected, and siΔNp73-transfected) compared to nonR-SKOV3 after induction of additional damage up to 72 h. Data represent the means ± SD from triplicate experiments. *p < 0.05, **p < 0.01, ***p < 0.001, ****p < 0.0001, NS not significant.

The *TP73* gene, as a member of the p53 family [20], encodes variant isoforms including a N-terminally truncated form known as ΔNp73 [21], which inhibits the activity of TAp73 (transcriptionally active p73) and p53 [22, 23]. It has been reported that ΔNp73 regulates the expression of the ABC family genes by suppressing p53 at the promoter level [24]. This led us to examine whether ΔNp73 acts as an upstream regulator of ABCB1 and ABCB1-mediated phenotypes conferred by resistance to carboplatin in p53^null^ ovarian cancer cells. CarR-SKOV3 showed a higher level of ΔNp73 compared to nonR cells at both mRNA and protein levels (Fig. 5A, B). Upon effective knockdown of ΔNp73 in carboplatin-resistant cells, ABCB1 expression was significantly decreased, displaying dramatically suppressed growth rates with reduced number of Ki67-positive cells compared to non-treated carR-SKOV3 (Fig. 5C–G and Supplementary Fig. 4A, B). Silencing of ΔNp73 in carR-SKOV3 rendered more vulnerability with additional DNA damage induced by high-dose carboplatin treatment in a time-dependent manner, exhibiting a similar level of the viable rate of ΔNp73-knockdown carR-SKOV3 to nonR-SKOV3 (Fig. 5H). This might imply that oncogenic ΔNP73 acts as an upstream regulator of ABCB1 and its suppression reverses the carboplatin-resistant phenotype in SKOV3.

### ABCB1 suppression re-sensitizes carboplatin-resistant SKOV3 via restoring attenuated DNA repair activity of G2/M-accumulated cells

**Fig. 6:**
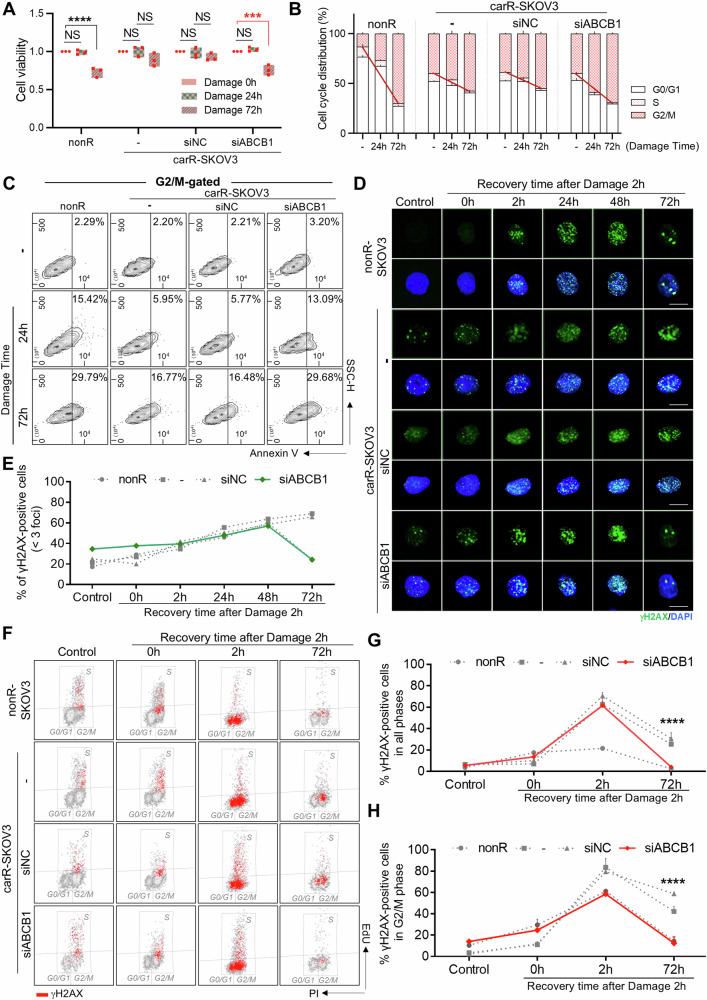
Re-sensitization of carboplatin-resistant SKOV3 by restoration of attenuated DNA repair function in G2/M-accumulated cells via ABCB1 inhibition. **A** Cell viability assay in carR-SKOV3 (non-treated, siNC-transfected, and siABCB1-transfected) compared to nonR-SKOV3 after induction of additional damage up to 72 h (carR-SKOV3 non-treated vs siABCB1-transfected: *p* = 0.0002, carR-SKOV3 non-treated vs nonR-SKOV3: *p* < 0.0001). Viability ratio was normalized to the value obtained from nonR cells. **B** Comparisons of cell cycle distribution in response to additional drug treatment in carR-SKOV3 (non-treated, siNC-transfected, and siABCB1-transfected) compared to nonR-SKOV3. The red line showed a gradient of time-dependent changes in G2/M-distributed cell proportion at the indicated time points. **C** Analysis of cell apoptosis occurred in G2/M-distributed carR-SKOV3 (non-treated, siNC-transfected, and siABCB1-transfected) compared to nonR-SKOV3 in response to the additional drug treatment. **D** Immunofluorescent staining of γH2AX (green) in carR-SKOV3 (non-treated, siNC-transfected, and siABCB1-transfected) compared to nonR-SKOV3 in response to 2 h of additional DNA damage induction and recovery time up to 72 h. Scale bar: 15 μm. DNA repair against genotoxic damage was calculated by counting cells (average of counted cell number: 64.6), which only includes γH2AX foci over 3. The proportion of recovered cells was visualized in a graph (**E**). **F** γH2AX detection in cell cycle progression incorporated with EdU staining by flow cytometry in carR-SKOV3 (non-treated, siNC-transfected, and siABCB1-transfected) compared to nonR-SKOV3 in response to recovery time up to 72 h after additional DNA damage induction. γH2AX positive expression in all phases (singlets-gated; **G**) and G2/M phase (**H**). Statistical analyses were conducted between the non-treated and siABCB1-transfected carR-SKOV3 groups. Data represent the means ± SD from triplicate experiments. *p < 0.05, **p < 0.01, ***p < 0.001, ****p < 0.0001, NS not significant.

We next asked whether ABCB1 inhibition-mediated reduction in stemness, especially found in the fraction of G2/M-accumulated SKOV3 cells, affected the status of drug resistance displayed in carR-SKOV3. To address this, cell viability and cell cycle were evaluated after drug treatment to induce additional damage. In carR-SKOV3 cells, the number of viable cells was significantly decreased after 72 h of extra high-dose carboplatin treatment via ABCB1 inhibition (0.75-fold), similar to nonR-SKOV3 (0.73-fold), whereas non-treated or siNC-transfected carR-SKOV3 cells showed little changes (Fig. 6A). Notably, no significant differences were observed between carR- and nonR-OVCAR3 regardless of *ABCB1* knockdown (Supplementary Fig. 5A). CarR-OVCAR3 rather exhibited significantly reduced viability and elevated apoptotic rates when treated with a combination of carboplatin and APR-246 [25, 26], a small molecule that reactivates mutant p53 in cancer cells to finally enhance apoptosis compared to sole carboplatin treatment (Supplementary Fig. 5B, C). Upon additional high-dose carboplatin treatment, G2/M-accumulated nonR-SKOV3 cells were increased in a time-dependent manner, showing a high degree of inclination, to stop the cell cycle progression and commence DNA repair or programmed cell death. Interestingly, a similar pattern was observed in siABCB1-carR-SKOV3 cells, whereas non-treated or siNC-transfected carR-SKOV3 showed a time-dependent G2/M accumulation with a lower gradient than nonR- or siABCB1-carR-SKOV3 (Fig. 6B). These findings were supported by changes in the number of Ki67-positive and Annexin V-positive cells in the G2/M-accumulated fraction (Figs. 4H, I and 6C). Our findings revealed that reduced stemness and proliferative capacity in G2/M-accumulated cells mediated by ABCB1 inhibition decreased the level of drug sensitivity in p53^null^ ovarian cancer cells, but not in p53-expressing cells, suggesting that the strategy to overcome acquired resistance to carboplatin might differ depending on the mutation status of the *TP53* gene.

We further examined whether the restored drug sensitivity induced by ABCB1 inhibition was mediated by the recovery of attenuated DNA repair activity in carR-SKOV3 compared to nonR-SKOV3 cells. Cells were transfected with siRNA to knock down *ABCB1*, and DNA damage was induced through treatment with high-dose carboplatin. The kinetics of DNA damage response and repair were assessed at 0 and 2 h of exposure to carboplatin and at post-treatment up to 72 h by measuring the formation and dissociation of DNA damage-induced γ-H2AX foci at DNA double-strand breaks (DSBs). NonR-SKOV3 cells showed recruitment of γ-H2AX at DSB sites at 2 h of recovery time, followed by 2 h of carboplatin-mediated DNA damage induction. The number of γ-H2AX foci was gradually increased up to 48 h of recovery time, and most of the foci disappeared at 72 h of recovery time, indicating that most of the damaged DNA was repaired during the recovery time (Fig. 6D, E). However, carR-SKOV3 displayed a high level of γ-H2AX foci even without DNA damage induction, as well as undissociated γ-H2AX foci at the DSB sites even at 72 h of recovery time, implying that carboplatin-mediated DNA damage remained unrepaired in acquired carboplatin-resistant SKOV3 cells. Upon ABCB1 inhibition in carR-SKOV3, the level of γ-H2AX foci formation at 72 h of recovery time was dramatically diminished, similar to that in nonR-SKOV3 (Fig. 6D, E). This has been supported by EdU and γ-H2AX co-labeling flow cytometry analyses (Fig. 6F–H). High-dose carboplatin treatment-induced γ-H2AX-positive signals were predominantly detected at non-replicating S and G2/M phases, and these appeared all over the cell cycle phases at 2 h of recovery time after extra DNA damage induction in all types of cells. Interestingly, γ-H2AX-positive cell fraction observed in carR-SKOV3 remained until 72 h of recovery time was found to be mostly distributed in the G2/M phase of the cell cycle, which was dramatically recovered by ABCB1 inhibition to the level observed in non-resistant cells. Moreover, siABCB1-transfected carR-SKOV3 showed morphological changes to more round-shaped cells, similar to nonR, after 72 h of recovery time followed by additional high-dose carboplatin treatment (Supplementary Fig. 5D). Therefore, these results collectively suggest that G2/M-accumulated unrepaired cells were responsible for attenuated DNA repair activity leading to drug resistance in p53^null^ ovarian cancer cells, which can be reversed by ABCB1 inhibition.

### Higher ABCB1 is associated with poor PFS and high resistance to platinum drugs in patients with ovarian cancer harboring p53^null^ mutations

**Fig. 7:**
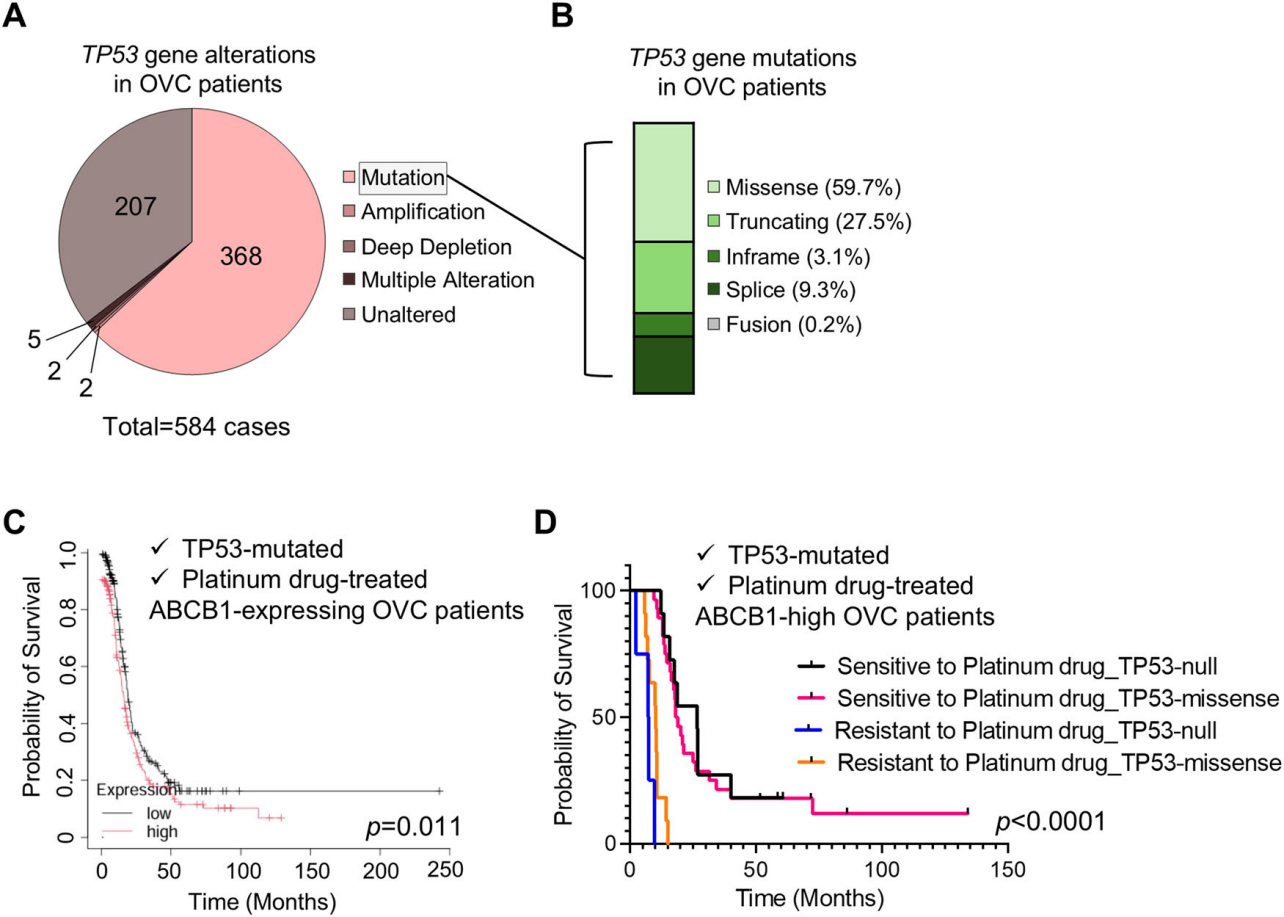
Clinical correlation between ABCB1 expression and PFS in ovarian cancer patients with platinum drug resistance. **A** *TP53* gene alterations in patients with ovarian cancer. **B** *TP53* mutation types in patients with ovarian cancer. **C** PFS analyses of platinum drug-treated ovarian cancer patients with *TP53* mutation depending on the status of ABCB1 expression (high vs low). **D** PFS analyses on the status of resistance to platinum drugs depending on the *TP53* mutation types (null vs missense) in ABCB1-high expressing ovarian cancer patients who were treated with platinum drugs.

To investigate the clinical relevance of ABCB1 expression status depending on the type of *TP53* mutations with progression free survival (PFS) rates mediated by acquired resistance to platinum-based drugs in patients with ovarian cancer, we assessed the data from human serous ovarian carcinoma samples in the cBioPortal and COSMIC databases for molecular alterations, gene expression, and treatment history with PFS [27–29]. The Cancer Genome Atlas (TCGA-PanCancer Atlas) ovarian serous carcinoma dataset showed that 67% (368/584 cases) of patients with ovarian cancer (total = 584 cases) retained the *TP53* gene mutations, including missense (59.7%: 228 cases), truncating (27.5%: 105 cases), in-frame (3.1%: 12 cases), splice (9.3%: 36 cases), and fusion (0.2%: one case) types (Fig. 7A, B). For consistency with our in vitro data using carboplatin-resistant SKOV3 and OVCAR3 cell lines, which harbored truncating (effectively as null) and missense mutations, respectively, only ovarian cancer samples with truncating or missense mutations in the *TP53* gene that were previously exposed to platinum drugs were assessed (Fig. 7C, D). Analyses of ovarian cancer cohorts in the National Center for Biotechnology Information (NCBI) Gene Expression Omnibus (https://www.ncbi.nlm.nih.gov/geo/) and the Genome Data Commons Data Portal (https://portal.gdc.cancer.gov/) [30] revealed that there is a significant correlation (*p* = 0.011) between the level of *ABCB1* gene expression and PFS in patients with p53 mutation-harboring ovarian cancer previously treated with platinum drugs, displaying a median survival of 19.13 months for the low ABCB1 expression cohort and 15.87 months for the high ABCB1-expressing group (Fig. 7C). This implies that patients with higher ABCB1 expression showed a shorter period of disease relapse. Furthermore, analyses using the TCGA ovarian cancer RNA-seq dataset presented via the UCSC Xena platform [31] for PFS in platinum drug-sensitive or -resistant groups with *TP53* null or -missense mutations displaying a high level of *ABCB1* expression revealed that patients sensitive to platinum drugs showed significantly longer PFS than those who were platinum drug-resistant. Moreover, platinum drug-resistant patients harboring p53^null^ mutations exhibited a shorter median PFS period (7.4 months) than those with p53^missense^ mutations (10.2 months), whereas platinum drug-sensitive groups showed a longer PFS in patients with p53^null^ mutations (26.7 months) than in those with p53^missense^ mutations (18.5 months) (Fig. 7D). These analyses indicate that the status of *ABCB1* gene expression has a larger impact on patients with ovarian cancer resistant to platinum drugs than on platinum drug-sensitive patients; in drug-resistant patients, a stronger correlation was observed in patients who retained p53^null^ mutations than in those groups with missense mutations in the *TP53* gene.

## Discussion

**Fig. 8:**
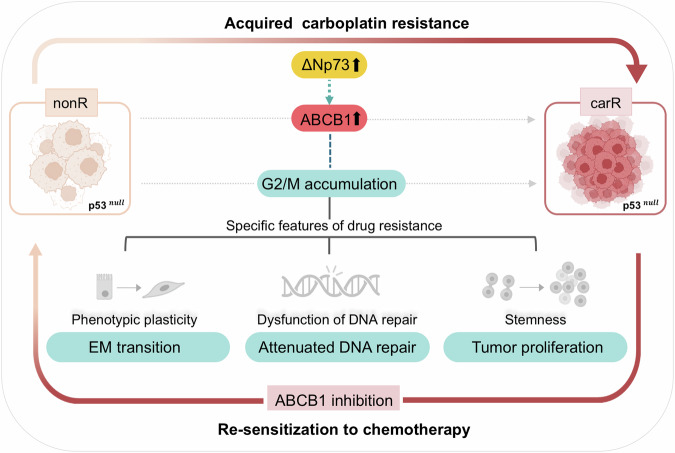
A schematic overview of molecular mechanisms underlying acquired resistance to carboplatin in p53^null^ human ovarian cancer.

This study demonstrated that acquired resistance to carboplatin in p53^null^ SKOV3 cells induced a significant portion of cells to accumulate in the G2/M phase of the cell cycle, displaying high levels of stemness marker expression with proliferative capacity, which we believe was reversed by inhibition of the ΔNp73-ABCB1 axis to the levels observed in non-resistant parental cells. ABCB1 suppression re-sensitized carboplatin-resistant cells to additional genotoxic stress by recovering DNA repair activity and lowering stemness-like features, especially in the G2/M-accumulated fraction. This suggests that the high levels of stemness and low levels of DNA repair activity exhibited in the G2/M-accumulated cell fraction may be responsible for the more aggressive and drug-resistant phenotypes displayed in p53^null^ SKOV3, but not in p53^R248Q^ OVCAR3 (Fig. 8).

Under normal conditions, cells exposed to excessive DNA damage induced by repeated genotoxic stress, such as prolonged drug treatments, are usually eliminated by the p53-mediated apoptosis process [32]. The loss of its apoptotic function contributes to cancer progression, proliferation, stemness, and metastasis, leading to the development of radio- and drug-resistance in cancer cells [33]. Many studies have shown that ovarian cancer cells with p53^null^ mutations (found in SKOV3), which mediate the complete absence of p53 expression and function, exhibit a worse prognosis than other types of p53-expressing mutation harboring ovarian cancers [34]. Loss of p53 function is strongly associated with high levels of multidrug resistance in neuroblastoma cells [35] and increased survival of progenitor stem cells in multiple myeloma cells [36]. Consistently, our findings showed that drug response and DNA repair activity were severely attenuated in acquired carboplatin-resistant SKOV3, where p53 was completely absent (Figs. 1 and 6). In addition, carR-SKOV3 cells were highly viable even in the presence of additional high-dose carboplatin treatment and displayed higher expression levels of stemness-related markers than nonR (Supplementary Fig. 1 and Fig. 1). The p53^R248Q^ mutation (displayed in OVCAR3) plays a gain-of-function role in many types of cancers, resulting in earlier tumor onset and reduced patient lifespan [37]. Depletion of p53^R248Q^ protein results in suppressed proliferation of cancer cells with reduced cell cycle progression affected by deregulated cell-extracellular matrix interactions in esophageal squamous cell carcinoma [37]. However, in breast and lung cancer cells, p53^R248Q^ decreases cell motility and invasiveness in a p53 transactivation-dependent manner [38], implicating p53^R248Q^-induced distinct phenotypic features in different cell types. Furthermore, p53^R248Q^ overexpression increased the sensitivity of EGFR and MDM2 inhibitor treatments in OVCAR3, which supports our findings that carR-OVCAR3 showed little differences in drug response and DNA repair activities compared to nonR-OVCAR3 with sole treatment of carboplatin even at a high concentration. OVCAR3 with carboplatin resistance was more responsive to combinatorial treatment with carboplatin and APR-246, implying that reactivation of mutant p53 in OVCAR3 is required to re-sensitize ovarian cancer cells harboring R248Q mutation to chemotherapy to trigger programmed cell death (Supplementary Figs. 1 and 5).

Uncontrolled cell cycle progression, which results in excessive cell proliferation and drug resistance, is considered a major driver of genomic instability, leading to tumorigenesis [39]. One of the primary cytotoxic responses to drugs is a reduction in the cell population of the G0/G1 phase and an arrest in the G2/M phase of the cell cycle, the latter of which guides damaged cells to the programmed cell death signaling pathway [40]. The G2/M phase checkpoint controls to prevent cells with damaged DNA from undergoing the subsequent mitosis process, which is mainly mediated by inhibition of the CDK1-cyclin B complex [41]. G2/M phase arrest increases the cytotoxicity of anti-cancer drugs in various types of cancer cells in vitro [40, 42]. In contrast, acquired resistance to temozolomide induced prolonged G2/M arrest in human glioma cells via a dysfunctional mismatch in the DNA repair system [43]. Moreover, triple-drug (cisplatin, docetaxel, and 5-fluorouracil)-resistant head and neck cancer cells exhibited a decreased number of cells in the G0/G1 phase and an increased accumulation of cells in the G2/M phase [44]. This implies that prolonged accumulation in the G2/M phase might be utilized as a critical mechanism to escape the apoptotic pathway and render cells drug-resistant by adopting and tolerating the damaged DNAs. The present study indicated that a significant proportion of carR-SKOV3 cells accumulated in the G2/M phase of the cell cycle compared to nonR cells, whereas little difference was found between carR- and nonR-OVCAR3 (Fig. 2). Interestingly, G2/M-arrested carR-SKOV3 cells exhibited higher levels of stemness-related markers than G2/M-arrested nonR cells (Fig. 2D), which was reversed by the ABCB1 inhibition (Fig. 4G). However, p53^R248Q^-OVCAR3 showed no significant differences between the carR- and nonR-groups (Fig. 2E). This might imply that the loss of p53 function mediated by p53^null^ mutations induces acquired resistance to carboplatin by accumulating cells in the G2/M phase of the cell cycle, rendering these cells with more stem-like features to evade the apoptotic pathway, which is not applicable for p53-expressing mutations harboring ovarian cancers.

Our interrogation of RNA-seq analyses of carR-SKOV3 compared to nonR-SKOV3 in the present study identified the *ABCB1* gene, which was involved in two gene ontology pathways: cell cycle and stem cell proliferation (Fig. 3 and Supplementary Fig. 3). We verified that the *ABCB1* expression pattern in SKOV3 cells was opposite to that found in OVCAR3 cells, suggesting that *ABCB1* may be a potential candidate for regulating the stemness of cells arrested in the G2/M phase and eventually carboplatin resistance in p53^null^ ovarian cancer. Consistent with the RNA-seq analyses, ABCB1 inhibition reduced the expression of stemness-related markers in G2/M-accumulated carR-SKOV3 cells without affecting the cell cycle progression, resulting in the re-sensitization of carboplatin-resistant cells to additional genotoxic stress to the levels of non-resistant parental cells (Fig. 4). Numerous studies have established a strong association between the expression of ABCB1, ATP-binding cassette (ABC) subfamily B member 1, and the development of multidrug resistance in cancer cells acquiring resistance to chemo-therapeutic drugs, including carboplatin and paclitaxel [45]. It has been reported that overexpression of ABCB1 is considered a major impediment to the effectiveness of chemotherapy in numerous cancers [46–48], which aligns with the findings presented in the present study (Figs. 4 and 6). Moreover, ABCB1 is regulated by *TP73* gene, which shares a high degree of structural homology with p53 and exhibits similar biological activities by controlling its transcriptional targets [49–52]. *TP73*, a member of the p53 family, is expressed in multiple variants. The full-length of TAp73 isoforms have tumor-suppressor potential, while certain variants show oncogenic properties. One such variant, ΔNp73, acts as a dominant-negative inhibitor of both TAp73 and wild-type p53, thereby inhibiting drug-induced apoptosis [24, 53, 54]. Additionally, ΔNp73 regulates the expression of genes associated with drug resistance, including *ABCB1* [55]. Consistent with these findings, our data demonstrate the ΔNp73-mediated regulation of ABCB1 involving the oncogenic biological events of carR-SKOV3 cells induced by upregulated ABCB1 (Fig. 5). This might imply that ΔNp73 acts as an upstream regulator of ABCB1 contributing to the development of chemo-resistant phenotypes in p53^null^ ovarian cancer cells. Our present study revealed that re-sensitization of carboplatin-resistant cells was mediated by the restoration of DNA repair activity accompanied by a significant reduction of stemness-like features especially in the G2/M-accumulated cell fraction via ABCB1 inhibition (Fig. 6). It might prevent numerous adverse effects affecting proliferating normal cells together with cancer cells that might be caused by direct targeting of cell cycle checkpoints to correct aberrant cell cycle progression occurred in various cancers [56].

Significantly, we demonstrated that acquired resistance to carboplatin in p53^null^ SKOV3 cells induced a significant portion of G2/M phase cell accumulation, where cells exhibited high levels of stemness-like features with proliferative capacity, which was effectively reversed by ABCB1 inhibition to the levels observed in non-resistant parental cells without affecting cell cycle progression. ABCB1 suppression re-sensitizes carboplatin-resistant cells to the additional genotoxic stress by recovering DNA repair activity and lowering their stemness, especially in the G2/M-accumulated fraction of cells, resulting in reduced proliferation and increased apoptosis. Notably, these findings were not applicable to p53^R248Q^-harboring ovarian cancer cells, suggesting that different molecular mechanisms underlie acquired drug resistance depending on the *TP53* mutation status. We propose ABCB1 as an effective molecular target for carboplatin-resistant ovarian cancers harboring p53^null^ mutations, which we uncovered could be used to increase the efficacy of conventional anti-cancer therapies, to develop more efficient combinatorial therapeutic interventions directed toward overcoming the chemoresistance and improving the survival rates in patients with ovarian cancer.

## Materials and methods

### Cell culture and generation of carboplatin-resistant cell lines

Human ovarian adenocarcinoma cell line SKOV3 and OVCAR3 were obtained from the Korea cell line bank (KCLB) and American Type Culture Collection (ATCC), respectively, and maintained in Roswell Park Memorial Institute (RPMI) 1640 medium supplemented with HEPES (SKOV3) or without HEPES (OVCAR3) containing 10% fetal bovine serum (FBS) and 1% penicillin-streptomycin (all from GIBCO, USA)_._ Carboplatin-resistant cell lines were generated according to an established protocol [16], in which a continued exposure to each concentration (2.5 μM for SKOV3 and 0.25 μM for OVCAR3) of carboplatin (S1215, Selleckchem, Germany) was conducted over 20 passages for approximately 6 months. Carboplatin was diluted in water to a final concentration of 25 mM at 25 °C to create a stock solution, which was then stored at −80 °C. The concentration for each cell type was determined by IC_50_ values described previously [17]. Carboplatin-resistant and non-resistant parental cells were named as carR- and nonR-, respectively.

### Cell proliferation assay

Cells were seeded in a 6-well plate (2 × 10^5^ cells/well) and observed for 48 h or 72 h. The proliferative capacity was evaluated by direct cell counting, and each growth rate was visualized as previously described [16].

### Cell viability and wound healing assay

Cells were seeded and maintained to 100% confluency in a 6-well plate. For the additional induction of DNA damage, 10-fold increased concentrations of carboplatin were administered to each group for 0 h, 24 h, and 48 h. In addition, APR-246 (Eprenetapopt; 5291-32-7; MedChemExpress, USA), purchased as a pre-diluted solution in DMSO at a concentration of 10 mM/mL and stored at −80 °C, was applied at the concentrations and duration as previously described [57]. For the evaluation of cell viability, direct cell counting was conducted. For the wound healing assay, a linear scratch was generated after starvation for 24 h. Gap distances of wound closure were measured at serial time points and quantified using Image J software (NIH, USA).

### Flow cytometry

#### Stemness profiling

Cells were harvested and fixed with 70% ethanol, subsequently resuspended in Fluorescence Activated Cell Sorting (FACS) buffer (0.5% bovine serum albumin (BSA) in 1× PBS). Single cell suspensions were incubated with APC-conjugated anti-CD133 (clone 7; BioLegend, USA), anti-LGR5 (SA222C5; BioLegend, USA), anti-CD117 (REA996; Miltenyi Biotec, USA), and FITC-conjugated anti-SOX2 (REA320; Miltenyi Biotec, USA) for 30 min. Cells were analyzed using Cyto-FLEX-Analyzer (Beckman Coulter, USA).

#### Cell cycle and apoptotic analysis

For cell cycle analysis, fixed cells were stained with 100 μg/ml of propidium iodide (PI) (Thermo, USA) in the presence of 0.1 μg/ml RNase A (Thermo, USA) for 30 min. For cell apoptotic analysis, cells were stained using the Annexin V-FITC apoptosis detection kit (BD Biosciences, USA) according to the manufacturer’s protocol. The level of apoptosis was calculated as the sum of the Annexin V-positive (early) and Annexin V/PI double-positive (late) fractions. Additionally, to quantify the cell apoptotic portion in G2/M phase, Vybrant™ DyeCycle Orange (Invitrogen, USA) and Annexin V-Pacific Blue (Invitrogen, USA) were adapted.

#### Cell cycle analysis and γH2AX detection with EdU incorporation

To visualize γH2AX expression with cell cycle progression, the Click-iT™ EdU Alexa Fluor™ 647 Flow Cytometry Assay Kit (Invitrogen, USA) was used according to the manufacturer’s instructions. Briefly, cells were incubated in a culture medium with 10 µM 5-ethynyl 2´-deoxyuridine (EdU) for 2 h and underwent a fixation and permeabilization process. The cells were then labeled with Azide 647 click detection cocktail and then stained with Alexa Fluor® 488 anti-H2A.X-Phospho (Ser139) antibody (BioLegend, USA) for 30 min. Finally, cells were stained with 100 μg/ml of PI in the presence of 0.1 μg/ml RNase A. All samples were run on a Cyto-FLEX-Analyzer (Beckman Coulter, USA) and data were analyzed by CytExpert software (Beckman Coulter, USA).

### Quantitative real-time reverse transcription polymerase chain reaction analysis

Total RNA extraction and reverse transcription to complementary DNAs were conducted as previously described [58]. Briefly, amplifications were run using a CFX Connect^TM^ Real-Time PCR Detection System (BioRad, USA). mRNA expressions were normalized to the housekeeping gene *ACTB*. Primer sequence pairs used in the experiments are shown in Supplementary Table 1.

### Immunoblotting analysis

Immunoblotting analysis was performed as previously described [58]. In brief, 20 μg of protein was separated and then transferred to polyvinylidene fluoride (PVDF) membranes (Millipore, USA). After blocking procedure, membranes were incubated with primary antibodies against p53 (7F5), phospho-p53 (E9Y4U; Ser15), PARP1 (46D11), cleaved-PARP1 (D64E10; Asp214), BCL-XL (54H6), phospho-cdc25c (63F9; Ser216), cyclin B1 (D5C10), ΔNp73 (38C674.2; Novus, USA), and β-actin (all from Cell signaling, USA), respectively, and finally visualized by ECL solution (Thermo, USA). Quantification of each protein expression compared to the loading control was conducted by using ImageJ software.

### RNA-seq analysis

To investigate the candidate genes, our previous data (GEO173579) were analyzed [16]. Differentially expressed genes (DEGs) were distinguished by a set for differences with fold-change (>2.0), *p*-value (<0.05), and absolute log2 normalized data (=0) between nonR and carR groups. Data mining was performed with ExDEGA v.1.6.5 software (ebiogen, South Korea) [59, 60]. Gene classification for gene ontology (GO) analysis was performed by Database for Annotation, Visualization and Integrated Discovery (DAVID; http://david.abcc.ncifcrf.gov/) [61], and the R package, ggplot2, was used for data visualization [62]. Moreover, gene expression analysis with specific GO terms was executed on the Molecular Signatures Database (MSigDB; https://www.gsea-msigdb.org/gsea/msigdb/human/search.jsp)-based gene collections and visualized with a heatmap using the Multiple Experiment Viewer (MeV) [63]. GSEA [64] was also performed to identify significantly enriched pathways in which the *ABCB1* gene is strongly involved.

### RNA silencing and transfection

For the transient knockdown of the ABCB1 gene, small interfering RNA (siRNA) targeting human *ABCB1* (Forward: ACAGAAUUAUGAAGAAGAGGU, Reverse: AACAGAGAUACCUCUUGAUA) and scrambled siRNA (IDT, USA) were used. In addition, *dNp73* siRNA was synthesized by Bioneer (Sense: GCGCCUACCAUGCUGUACGUC, Antisense: GACGUACAGCAUGGUAGGCGC) and applied as instructed, along with AccuTarget™ Negative Control siRNA. Cells were transfected using Lipofectamine 3000 (Thermo, USA) according to the manufacturer’s protocol.

### Immunofluorescence and microscopy

Cells were seeded on cover glasses in a 24-well plate, fixed with 4% paraformaldehyde for 13 min, and permeabilized with 0.5% Triton X-100 solution for 15 min at room temperature. Subsequently, cells were blocked with 5% BSA in 1× PBS with 0.5% Triton X-100 for 1 h, and incubated with γH2AX (Millipore, USA) overnight. Cells were mounted with Vectashield (Vector Laboratories, USA) containing 4′,6-diamidino-2-phenylindole (DAPI). Images were captured by a Zeiss 510 microscope (Carl Zeiss, Germany). γH2AX-stained dots were counted and quantified using ImageJ.

### Public data analysis

GEO, including GSE83440, GSE98559, and GSE148003 datasets (comparing cisplatin sensitive vs cisplatin resistant SKOV3 cells), were utilized for additional validation through correlation analysis. Raw read count and gene length data for the targeted *ABCB1* gene were extracted, calculated, and subsequently normalized to transcripts per million (TPM) values, using R version 4.1.1 software. Clinical *TP53* mutation data for human serous ovarian adenocarcinoma (TCGA, PanCancer Atlas, 585 samples) were downloaded from cBioPortal and the COSMIC database [27–29, 65, 66]. For the *ABCB1* gene expression data, RNA-seq V2 RESM data set and the progression-free interval (months) in ovarian cancer patients were downloaded from the UCSC Xena website [31]. Furthermore, Kaplan-Meier analyses for the correlation between ABCB1 expression and progression-free survival (PFS) in *TP53* mutation harboring ovarian cancer patients treated with platinum drugs were performed using the KM plotter, available at https://kmplot.com/analysis/. Survival analysis, focusing on PFS, was performed using Affy ID 209993_at, which corresponds to the probe for ABCB1. The analysis employed an auto-selected best cut-off based on the percentile method. The follow-up threshold was set to include all available data. A cut-off value of 43 was used in the analysis, which falls within the range of expression values for the probe (1–530). This study specifically included samples with serous ovarian cancer histology, spanning all stages and grades, with a focus on those harboring TP53 mutations. Only patients who had undergone debulking surgery were included, and the chemotherapy regimen was restricted to platinum-based agents. The hazard ratio (HR) value for this analysis was 1.35. For specified patients, the ABCB1 expression values ranged from 1 to 530, with a median value of 43. Accordingly, patients with ABCB1 expression values below 43 were classified into the ‘low expression group’, while those with expression values of 43 or higher were classified into the ‘high expression group’. The dataset utilized for this analysis encompassed all available samples, with array quality control measures in place to exclude any biased arrays. Additionally, the user-selected probe set allowed for a targeted evaluation, enhancing the robustness and precision of the results in assessing PFS outcomes.

### Statistical analysis

Comparison groups were analyzed with an unpaired t-test for parametric distributions. For multiple comparisons, the ordinary one-way ANOVA analysis with Dunnett’s multiple comparison test or two-way ANOVA analysis with Sidak’s multiple comparisons test was performed. For all cases, a *p*-value that was <0.05 was considered statistically significant (*p* < 0.05(*), *p* < 0.01(**), *p* < 0.001(***) and *p* < 0.0001(****)).

## Supplementary information

Original Western blot file

Supplementary figures and table

## Data Availability

The authors declare that all other data supporting the findings of this study are available within the article and its Supplementary information files. Please contact the corresponding author for data on reasonable request.
