# Supplementary figures and images for: ABCB1 confers resistance to carboplatin by accumulating stem-like cells in the G2/M phase of the cell cycle in p53null ovarian cancer
Source: Cell Death Discov. 2025 Apr 2;11:132. doi: 10.1038/s41420-025-02435-7 (PMC11965561; doi:10.1038/s41420-025-02435-7)

## Slide 1
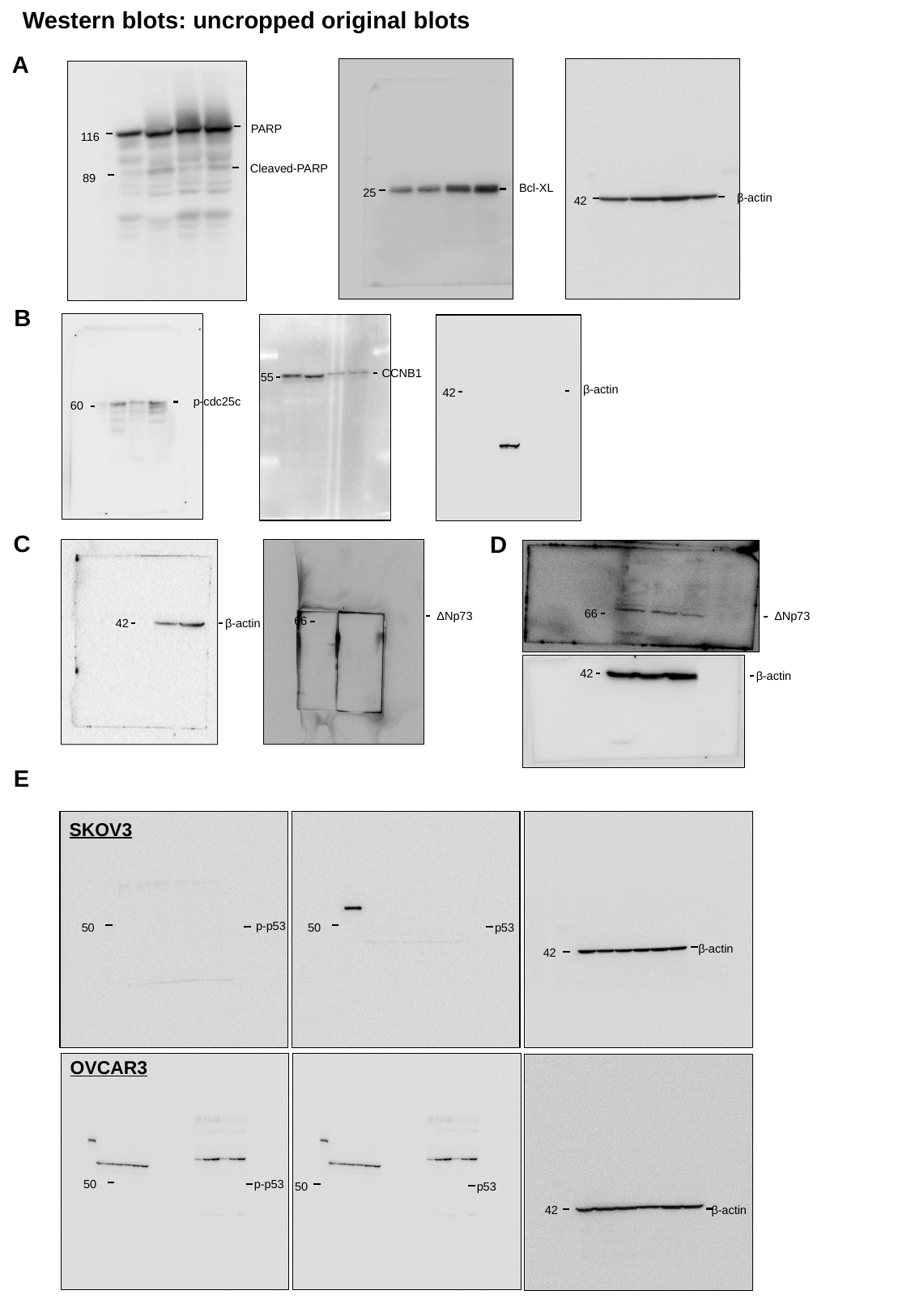

Supplement: Supplementary file 1 — Original Western blot file [file 41420_2025_2435_MOESM1_ESM.pptx]
