# Supplementary material for: ABCB1 confers resistance to carboplatin by accumulating stem-like cells in the G2/M phase of the cell cycle in p53null ovarian cancer
Source: Cell Death Discov. 2025 Apr 2;11:132. doi: 10.1038/s41420-025-02435-7 (PMC11965561; doi:10.1038/s41420-025-02435-7)
Supplement: Supplementary file 2 — Supplementary figures and table [file 41420_2025_2435_MOESM2_ESM.docx]

**ABCB1 confers resistance to carboplatin by accumulating stem-like cells in the G2/M phase of the cell cycle in p53^null^ ovarian cancer**

Danbi Lee^1,#^, Hyun-Seok Jeong^1,#^, Sun-Young Hwang^1^, Yu-Gyeong Lee^1^, Youn-Jung Kang^2,^*

**Affiliations**

1. Department of Biomedical Science, School of Life Science, CHA University, 335 Pangyo-ro, Bundang-gu, Seongnam-si, Gyeonggi-do, South Korea,13448
2. Department of Biochemistry, Research Institute for Basic Medical Science, School of Medicine, CHA University, 335 Pangyo-ro, Bundang-gu, Seongnam-si, Gyeonggi-do, South Korea,13448

**^#^**These authors contributed equally to this work

*Author of correspondence (yjkang@cha.ac.kr)

**Supplementary Fig. 1** **(A)** Immunoblotting analyses of p53 and phospho-p53 in carR-SKOV3 and -OVCAR3 compared to nonR groups, respectively, in response to additional damage induction up to 72 h. Loading control; β-actin. **(B)** QRT-PCR analyses of epithelial-mesenchymal transition-related genes (*CDH1, VIM*, and *CDH2*) in carR-SKOV3 and -OVCAR3 compared to nonR groups, respectively (*CDH1*; carR-SKOV3 vs. nonR-SKOV3; *p< 0.0001,* carR-OVCAR VS. nonR-OVCAR3; *p< 0.0001, VIM*; carR-SKOV3 vs. nonR-SKOV3; *p=0.066,* carR-OVCAR VS. nonR-OVCAR3; *p=0.534, CDH2*; carR-SKOV3 vs. nonR-SKOV3; *p=0.034,* carR-OVCAR VS. nonR-OVCAR3; *p=0.148*). **(C)** Cell proliferation assays in carR-SKOV3 and -OVCAR3 compared to nonR groups, respectively (carR-SKOV3 vs. nonR-SKOV3; *p=0.014*). **(D-E)** Representative images of wound healing assay in carR-SKOV3 and -OVCAR3 compared to nonR groups, respectively, with or without additional damages. Area of wound closures were normalized to each nonR group (carR-SKOV3 vs. nonR-SKOV3; *p=0.0008*, carR-SKOV3 vs. nonR-SKOV3 after additional damage; *p=0.014* and carR-OVCAR VS. nonR-OVCAR3; *p=0.012*). Scale bar; 50 μm. **(F)** Cell viability assays in carR-SKOV3 and -OVCAR3 compared to nonR groups, respectively, in response to additional damage induction up to 72 h (carR-SKOV3 vs. nonR-SKOV3; *p=0.0059* and carR-OVCAR VS. nonR-OVCAR3; *p=0.0315*). (NS, p>0.05; *, p<0.05; **, p<0.01; ***, p<0.001; ****, p<0.0001).

**Supplementary Fig. 2** Quantified intensities of immunoblotting bands showing phospho-cdc25c **(A)** and Cyclin B1 **(B)** in carR-SKOV3 and -OVCAR3 compared to nonR groups, respectively, shown in **Figure 2C** (**p-cdc25c:** carR-SKOV3 vs. nonR-SKOV3; *p=0.004*, carR- OVCAR3 vs. nonR- OVCAR3; *p=* *0.008,* **Cyclin B1:** carR-SKOV3 vs. nonR-SKOV3; *p<0.001*, carR- OVCAR3 vs. nonR- OVCAR3; *p=0.334*). Data represents the means ± SD from triplicate experiments. *; *p<0.05*, **; *p<0.01*, ***; *p<0.001*, ****; *p<0.0001*, NS; not significant.


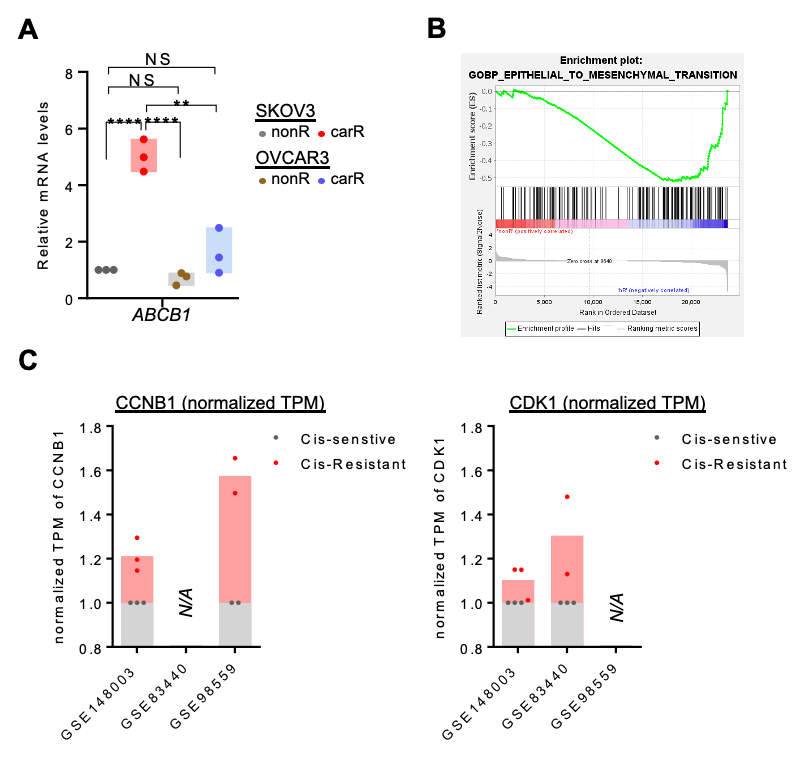


**Supplementary Fig. 3** **(A)** Relative mRNA expressions of *ABCB1* in carR-SKOV3 and -OVCAR3 compared to nonR groups, respectively. **(B)** GSEA analysis of EMT (GO:0001837) enrichment in carR-SKOV3 compared to nonR-SKOV3. **(C)** Analysis of datasets (GSE83440, GSE98559, and GSE148003) including upregulated expression of cell cycle related-genes (*CCNB1* and *CDK1*) in cisplatin-resistant SKOV3 compared to cisplatin-sensitive SKOV3. Data were quantified by normalized TPM levels of ABCB1 in each group.

**Supplementary Fig. 4** **(A)** Quantified intensities of immunoblotting bands showing ΔNP73 in nonR- and carR-SKOV3, shown in **Figure 5B**, **(B)** Quantified intensities of immunoblotting bands showing validation of *Δ*Np73 silencing in carR-SKOV3 compared to non-treated and negative control (NC) transfected-groups, shown in **Figure 5C.**

**Supplementary Fig. 5** **(A)** Cell viability assays in carR-OVCAR3 compared to nonR groups, respectively, in response to additional damage induction up to 72 h. **(B)** Cell viability assays were conducted by cell count in carR-OVCAR3 at 20 h post treatment with each combination of APR-246 and carboplatin. Statistical analyses were conducted between non-treated car-OVACR3 and other groups (nonR vs. carR; *p=0.302*, carR vs. carR+2.5 μm carboplatin; *p=0.040,* carR vs. carR+2.5 μm carboplatin+2 μm APR-246; *p=0.005*, carR vs. carR+2.5 μm carboplatin+5 μm APR-246; *p=0.018*, carR vs. carR+2.5 μm carboplatin+10 μm APR-246; *p=0.012*, carR vs. carR+2.5 μm carboplatin+20 μm APR-246; *p=0.007*, carR vs. carR+2.5 μm carboplatin+40 μm APR-246; *p=0.006).* **(C)** Annexin V-FITC/PI staining by flow cytometric analysis. Statistical analyses were conducted between non-treated car-OVACR3 and other groups (nonR vs. carR; *p=0.391*, carR vs. carR+2.5 μm carboplatin; *p=0.090,* carR vs. carR+2.5 μm carboplatin+2 μm APR-246; *p<0.001*, carR vs. carR+2.5 μm carboplatin+5 μm APR-246; *p<0.001*, carR vs. carR+2.5 μm carboplatin+10 μm APR-246; *p<0.001)*. Data represents the means ± SD from triplicate experiments. *; *p<0.05*, **; *p<0.01*, ***; *p<0.001*, ****; *p<0.0001*, NS; not significant. **(D)** Morphological changes in carR-SKOV3 (non-transfected-, siNC-transfected- and siABCB1-transfected-) compared to nonR-SKOV3 after inducing of additional damage prior to recovery time up to 72 h. Scale bar; 25 μm, 100 μm (magnified images in the lowest panel).

**Supplementary Fig. 6** Whole immunoblot shown in Fig. 1D **(A)**, Fig. 2C **(B)**, Fig. 5B **(C), Fig. 5C (D),** and Supplementary Fig. 1A **(E)**

| Species | Gene | Direction | Sequence |
| --- | --- | --- | --- |
| Human | *ACTB* | Forward | CATGTACGTTGCTATCCAGGC |
|  |  | Reverse | GCCTTAATGTCACGCACGAT |
|  | *BAK* | Forward | ATGGTCACCTTACCTCTGCAA |
|  |  | Reverse | TCATAGCGTCGGTTGATGTCG |
|  | *BAX* | Forward | CCCGAGAGGTCTTTTTCCGAG |
|  |  | Reverse | CCAGCCCATGATGGTTCTGAT |
|  | *CYTC* | Forward | GTGCCACACCGTTGAAAAG |
|  |  | Reverse | AGTGTATCCTCTCCCCAGATG |
|  | *BCL2L1* | Forward | GCATATCAGAGCTTTGAACAG |
|  |  | Reverse | GAAGGAGAAAAAGGCCACAAT |
|  | *ABCB1* | Forward | GGGAGCTTAACACCCGACTTA |
|  |  | Reverse | GCCAAAATCACAAGGGTTAGCTT |
|  | *ΔNp73* | Forward | CGCCTACCATGCTGTACGTC |
|  |  | Reverse | GTGCTGGACTGCTGGAAAGT |

**Supplementary Table 1. Primer sequence pairs used for QRT-PCR**
